# How to Compare the Ion Selectivity of Smart Nanopores/Membranes

**DOI:** 10.34133/research.0506

**Published:** 2024-10-25

**Authors:** Huma Bhatti, Yi-Lun Ying, Yi-Tao Long

**Affiliations:** Molecular Sensing and Imaging Center, School of Chemistry and Chemical Engineering, Nanjing University, Nanjing 210023, China.

## Abstract

Ion selectivity is a fundamental feature for designing advanced nanopores/channel systems, for example, biosensors or selectively permeable membranes. Comparison between different studies is a way to find and design the nanopore/membrane with pronounced selectivity. However, there is a huge hurdle in comparing the ion selectivities between studies, resulting from different equations from diverse scopes of science. Here, the authors from *“*Addressing challenges in ion-selectivity characterization in nanopores*”* emphasized the misinterpretation of the traditionally used Nernst and Goldman–Hodgkin–Katz equations in the previous literature and suggested the use of uniform criteria to overcome this ambiguity. We highlight the potential future applications of using uniform criteria in describing ion selectivity, which is beneficial to developing a massive AI-based databank. This databank would be advantageous for predicting and designing the ion selectivity of nanopores/nanomaterials in question for various applications in biological and material sciences.

Ion selectivity refers to the ability of a material (such as a membrane) or a device (ion channel or nanopore) to preferentially allow certain ions to pass through while restricting others [1]. This selective transport of ions is based on their size, charge, and chemical interactions with the materials they are translocating through. This property is essential for the functionality of various natural and engineered systems, including biological ion channels, water purification membranes, ion-selective electrodes, and energy storage devices. In biological channels, ion selectivity is indispensable to the proper functioning of cells and organisms. The selective nature of these channels allows cells to perform complex and highly regulated physiological processes, such as maintaining membrane potential, signal transduction, cellular homeostasis, muscle contraction, metabolic control, and hormone secretion, highlighting the critical importance of ion selectivity in biology [2]. Similarly, ion selectivity is imperative to synthetic systems such as water desalination, ion separation, osmotic energy conversions, and solid-state nanopores [3–6]. Therefore, understanding and controlling ion selectivity is crucial for advancements in medicine, environmental science, and energy technology. Driven by advancements in material science and chemical engineering, various research groups have made substantial strides in developing membranes and pore-forming materials with improved ion selectivity, by tailoring the physical and chemical properties of membranes, leading to better performance in a wide range of practical applications (Figure).

**Figure.**
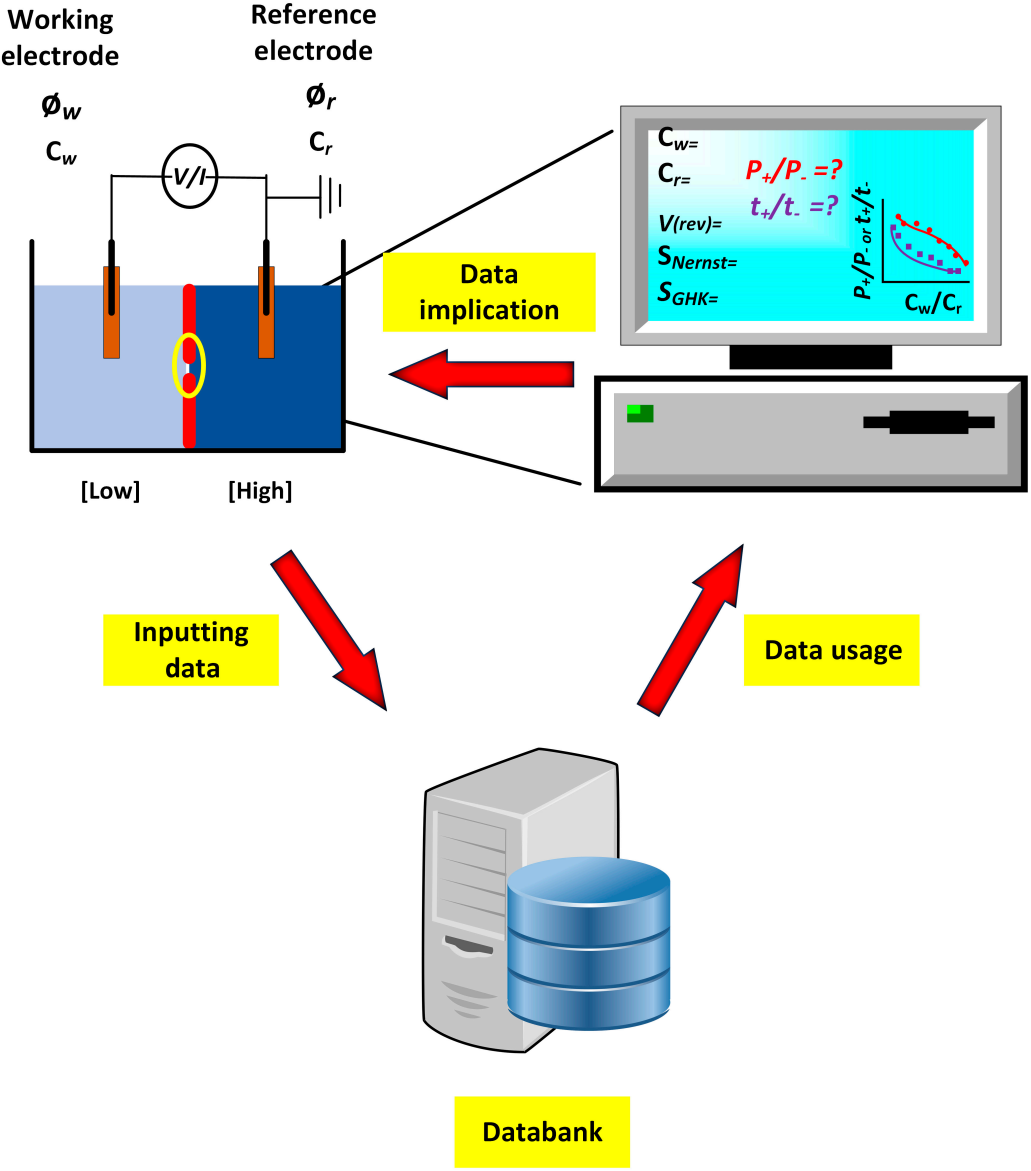
Schematic illustration for the future of electrode convention toward the development of ion-selectivity AI databank.

Traditionally, ion selectivity has been calculated by using 2 well-known equations, which are (a) the Poison–Nernst–Planck equation [7] and (b) the Goldman–Hodgkin–Katz (GHK) equation [8,9], by employing the transmembrane potential (at zero current) under a concentration gradient. The GHK equation is accustomed to determining the ion selectivity of biological channels and biosensor devices, whereas the Nernst equation has been adapted frequently in electrochemical and material sciences. The GHK equation originated from the Nernst equation, but the assumptions behind these 2 equations are dissimilar. The Nernst equation assumes the electrical neutrality of the solution system, whereas the Goldman equation undertakes that the electric field is constant; both equations are believed to possess constant permeabilities [7–9]. Because of different assumptions, the ion selectivity calculated from the Nernst and GHK equations is different. Therefore, it is irrational to compare the ion selectivity between studies from diverse domains of science using either of the 2 equations. Equations 1 and 2 represent the traditionally used Nernst and GHK equations.

$$\text{Nernst}:\;\left|V_{\mathrm{rev}}\right|=\left|\emptyset_{\beta }-\emptyset_{\alpha }\right|=\frac{\mathrm{RT}}{F}\;\left(1-2t_{\pm }\right)\ln\frac{a_{\beta }}{a_{\alpha }}$$ (1)

$$\text{GHK}:\;\left|V_{\mathrm{rev}}\right|=\left|\emptyset_{\beta }-\emptyset_{\alpha }\right|=\frac{\mathrm{RT}}{F}\ln\;\left(\frac{P_{\pm }C_{\alpha }\;+\;P_{\mp }C_{\beta }\;}{P_{\pm }C_{\beta }\;+\;P_{\mp }C_{\alpha }}\right)$$ (2)

To make it comparable yet logical, Zhang et al. [10] addressed the 2 main challenges to determining ion selectivity, both qualitatively and quantitatively. The first challenge modified the idea that, for a long time, the Nernst and GHK equations have been used but did not establish the definition of the 2 electrodes and arbitrarily were taken as *α* and *β* in the measurements, so it was hard to identify if *|Vrev|* was obtained from the *|ϕα − ϕβ| or |ϕβ − ϕα|* electrochemical system, where *ϕ* represents the electric potential. This is more commonly practiced in material science where each study independently assigns the electrodes as *α* and *β*. Therefore, using the absolute values, the direction (sign) of the measured electric potential, and hence the ion for which the membrane is selective, is inexplicable. To circumvent the first challenge, the study [10] proposed that a clear definition of electrodes as working and reference electrodes (instead of *α* and *β*) is a prerequisite in determining the orientation of the electric field and hence the sign of reversal potential for determining and comparing ion selectivity qualitatively. The placement of low and high concentrations of the solutions must also be defined as *cw* and *cr* concerning their electrodes. In essence, uniform criteria are mandatory for comparison between different reports.

After defining electrodes, the study [10] emphasizes that the concentration ratio of solutions is pivotal to comparing ion selectivity quantitatively, resulting from the Nernst and GHK equations. To eradicate this ambiguity, they performed simulations to understand the nanopore’s physicochemical properties, the surface charge sign, and the magnitude of ion selectivity, which will help validate the design and make further improvements. Also, the ideal selectivity (S*_ideal_*) of the nanopore was calculated from simulations that are thought to be constant throughout the nanopore. However, it is hard to determine S*_ideal_* for nanopores because of the ion concentration polarization effect in ion-selective nanopores [11]. Therefore, the authors configured that the selectivities obtained from both equations are the magnitudes between the nanopore's ideal selectivities at low and high concentrations in the 2 chambers. Furthermore, under a high concentration ratio across the transmembrane, the selectivity is considered as an effective selectivity, which is the weighted averages of local selectivities (selectivities at a given position) in the nanopores/membranes. Alternatively, if a low concentration ratio of *cw/cr* is maintained, the magnitude of ion selectivity stemming from 2 different equations may come to a point where they coincide [10]. Thus, it is recommended to use a low concentration ratio if it does not affect the signal-to-noise ratio of the detecting system. Conclusively, this study suggests that the researchers should present both the Nernst and GHK selectivity including solutions’ concentrations used in both the chambers and *V_(rev)_* with its sign in their studies for an unbiased comparison between nanopores/membranes.

To extend their study, it is encouraged that all researchers should adopt the protocols proposed by Zhang et al. and use the uniform criteria to establish an ion-selectivity databank that will put forward the design of nanopores/membranes from multiple domains of science such as biosensors, material chemistry, or membrane protein engineering. In this databank, the ion selectivities must be represented by using both the Nernst and GHK equations, and the description of solution compositions and concentrations at both the working and reference electrodes’ sides will be mentioned. Additionally, by using simulations and artificial intelligence (AI), this picture can be broadened even more for the precise quantification of ion selectivity, which is imperative for designing and improving nanopores/channel systems.

We anticipate that this databank should also contain the data for the selectivities of previously designed nanopores/materials calculated from either the Nernst or GHK equations to evaluate them on the uniform criteria proposed by the authors. In this way, we can revisit the nanopores/channels’ selectivity appropriately, and massive data for ion selectivity can be obtained. These data will fill the gaps by answering the effects of solution composition and transmembrane concentration ratio on the ion selectivity of the nanopores/materials in question. Moreover, this databank will also help guide our understanding of the selectivity of nanopores by employing different solution compositions to the nanopores already known. Consequently, an AI-based databank for ion selectivity would be updated each time a new solution composition is attempted and a great information portal would be formed to help understand the physicochemistry of the nanopores/materials. Hence, the desired ion selectivity can be achieved or predicted by using the databank for its diverse roles in medicine, material engineering, and other applications.
